# Case Report: Doege–Potter syndrome: a giant solitary fibrous pleural tumor causing severe hypoglycemia

**DOI:** 10.3389/fonc.2025.1550343

**Published:** 2025-06-04

**Authors:** Christelle De Vico, Carlo Tappero, Enzo Fontana, Jon Andri Lutz, Benoît Rouiller

**Affiliations:** ^1^ Unit of Thoracic Surgery, Department of Surgery, Fribourg Cantonal Hospital, Fribourg, Switzerland; ^2^ Faculty of Science and Medicine, University of Fribourg, Fribourg, Switzerland; ^3^ Unit of Diagnostic and Interventional Radiology, Department of Radiology, Fribourg Cantonal Hospital, Fribourg, Switzerland; ^4^ Unit of Diabetes and Endocrine, Department of Internal Medicine, Fribourg Cantonal Hospital, Fribourg, Switzerland

**Keywords:** non-islet-cell tumor hypoglycemia, NICTH, solitary fibrous tumor of the pleura, PSFT, Doege-Potter syndrome

## Abstract

Doege–Potter syndrome or non-islet cell tumor hypoglycemia (NICTH) is a rare entity usually due to a solitary fibrous tumor of the pleura (pSFT). The diagnosis of NICTH is challenging because of its non-specific clinical presentation and the rarity of pSFT. We present a case report of a 44-year-old woman diagnosed with NICTH to demonstrate the difficulty of establishing this diagnosis and the importance of preoperative preparation based on the characteristics of the lesion. We performed an embolization followed by an en bloc resection with diaphragmatic patch and lung wedge resection, and we subsequently reconstructed the diaphragm with Prolene mesh through a left hemiclamshell incision. The postoperative course was favorable, particularly the resolution of hypoglycemia, which was concordant with the diagnosis. Pathological examination revealed a pSFT with clean margins and no features of malignancy detected by immunohistochemistry. This case report highlights the fact that diagnosing Doege–Potter syndrome is not always easy and often takes time after the patient’s initial presentation, the importance of preoperative planning, and the benefits of preoperative embolization of the arterial feeder.

## Introduction

Doege–Potter syndrome or non-islet cell tumor hypoglycemia (NICTH) is a rare entity induced by paraneoplastic production of big insulin-like growth factor 2, causing hypoglycemia, most often due to a solitary fibrous tumor of the pleura (pSFT). Solitary fibrous tumors originate from mesenchymal cells (1). Theoretically, these tumors can arise from any connective tissue of the body (2, 3), but in most cases, they emerge from the pleura (1). They affect the visceral pleura more often than the parietal. pSFT occurs at any age but commonly between 40 and 60 years and has an incidence of 2.8 per 100,000 persons per year (2). The distribution between sexes is equal (4), and features of malignancy are found in approximately 20% of cases (1). Complete excision of the tumor is the treatment of choice. Minimal invasive or open access depends on tumor localization and size as well as the surgeon’s preference (4). There are insufficient data on the utility of adjuvant radiotherapy and chemotherapy (2, 7) and neo-adjuvant therapy (2). For giant tumors, defined as bigger than 15 cm or occupying 40% of the hemithorax, preoperative embolization may be useful to minimize blood loss (5, 7). Blood supply is provided principally from intercostal, internal mammary, or bronchial arteries, with a vascular pedicle present in 50% of cases (2, 6). Less frequently, the blood supply arises from the aorta or the celiac trunk (2, 5, 6). The histology reveals spindle cells with round nuclei immersed in a rich stroma of collagen fibers (7). The microscopic features indicating malignant potential are high mitotic activity, high cellularity, pleomorphism, and the presence of necrosis. The macroscopic and clinical features raising suspicion of malignancy are size (2), extrapleural localization (8), and NICTH (8). The recurrence rate is approximately 5%–17% for benign pSFTs and 14%–54% for the malignant ones; recurrence occurs principally in the first 2 years after resection (2). A CT scan every 6 months during the first 2 years after resection and then yearly is recommended as follow-up (2).

Depending on the size of the pSFT, symptoms such as dyspnea, cough, pleuritic chest pain, shortness of breath, fever, and weight loss can occur from mass effect (8). The diagnosis of NICTH is challenging because of its non-specific clinical presentation and the rarity of pSFT.

Our patient was a 44-year-old woman with no prior medical history who initially consulted the emergency department of our hospital with an acute confusional state. She was first hospitalized in the department of internal medicine and discharged after 4 days, her symptoms having resolved without a definitive diagnosis. A few weeks later, she had to be admitted to the intensive care unit for severe hypoglycemia.

An endocrinologic outpatient workup showed suppressed insulin secretion associated with low plasma glucose. Abdominal and thoracic CT scans revealed a large (15-cm) tumor of the right chest, suggesting pSFT, and the suspicion of Doege–Potter syndrome was suggested. The low IGF-1 value (30.4 μg/L) made the diagnosis even more likely. The case was discussed at the thoracic oncology board, and the indication for surgery was confirmed on the basis of the morphologic and radiological features and clinical history that were highly suggestive of the diagnosis.

The endocrinologic outpatient workup excluded other diagnoses explaining the symptoms and the low blood glucose levels. The differential diagnosis included other tumors, which were excluded by the abdominal chest CT scan. Moreover, peptide C was low (38 pmol/L), making the diagnosis of insulinoma unlikely. With low serum insulin (<2 mlU/L) and anti-insulin antibodies negative (<0.4 U/mL), the diagnosis of exogenous insulin production was also unlikely. The patient was taking no new medication and had no history of alcohol abuse that would explain the clinical presentation. She had undergone no abdominal surgery. Blood tests showed no renal, cardiac, or hepatic organ insufficiency, nor adrenal insufficiency (Adreno CorticoTropic Hormone (ACTH) 7.3 pmol/L, cortisol at 8 hours 419 nmol/L). This left NICTH as the remaining diagnosis, as described above.

## Method

**Figure 1:**
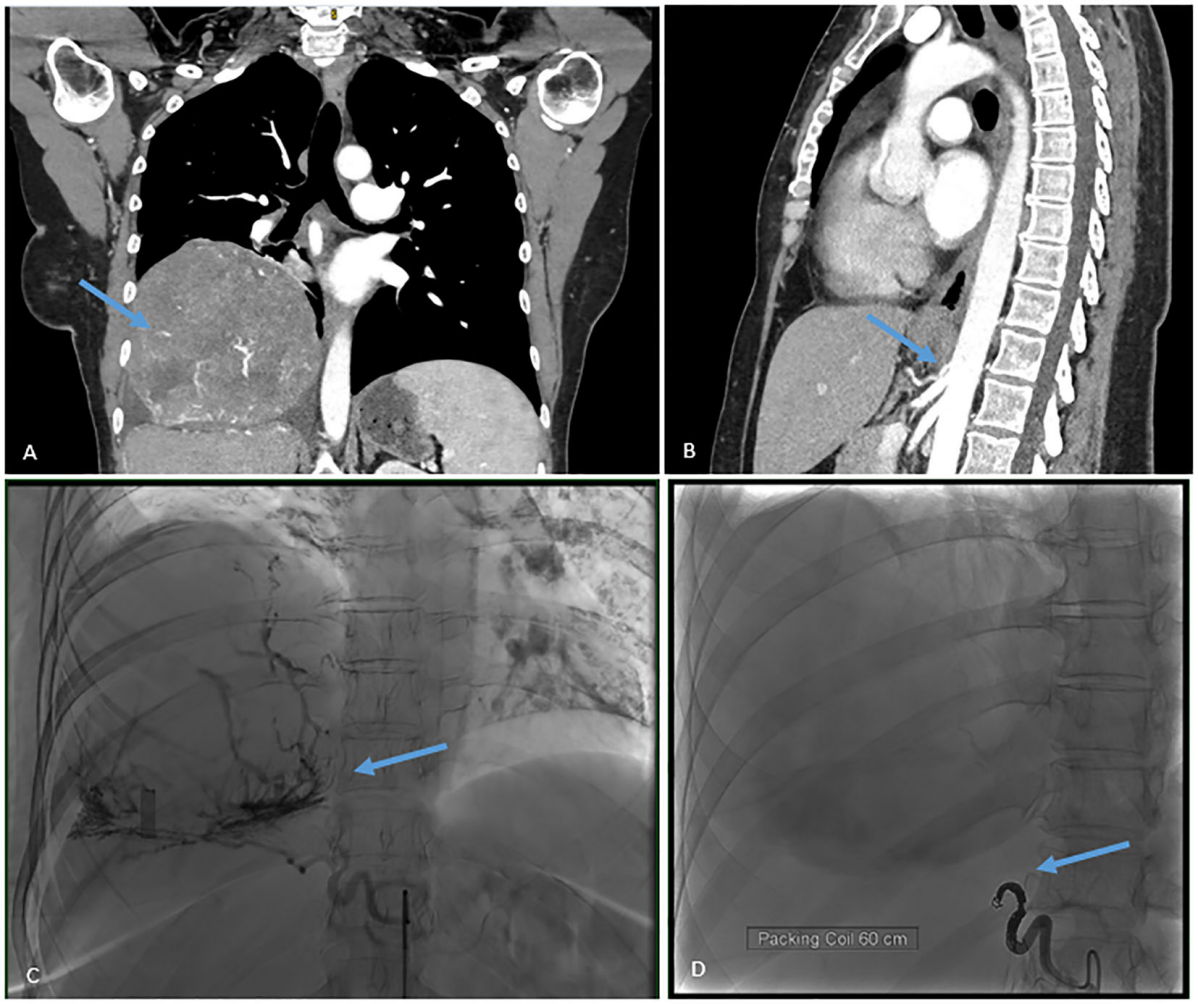
**(A)** CT scan showing the tumor. **(B)** CT scan showing the vascular pedicle arising directly from the aorta. **(C)** Pre-operative embolization before coiling. **(D)** Pre-operative embolization after coiling.

**Figure 2:**
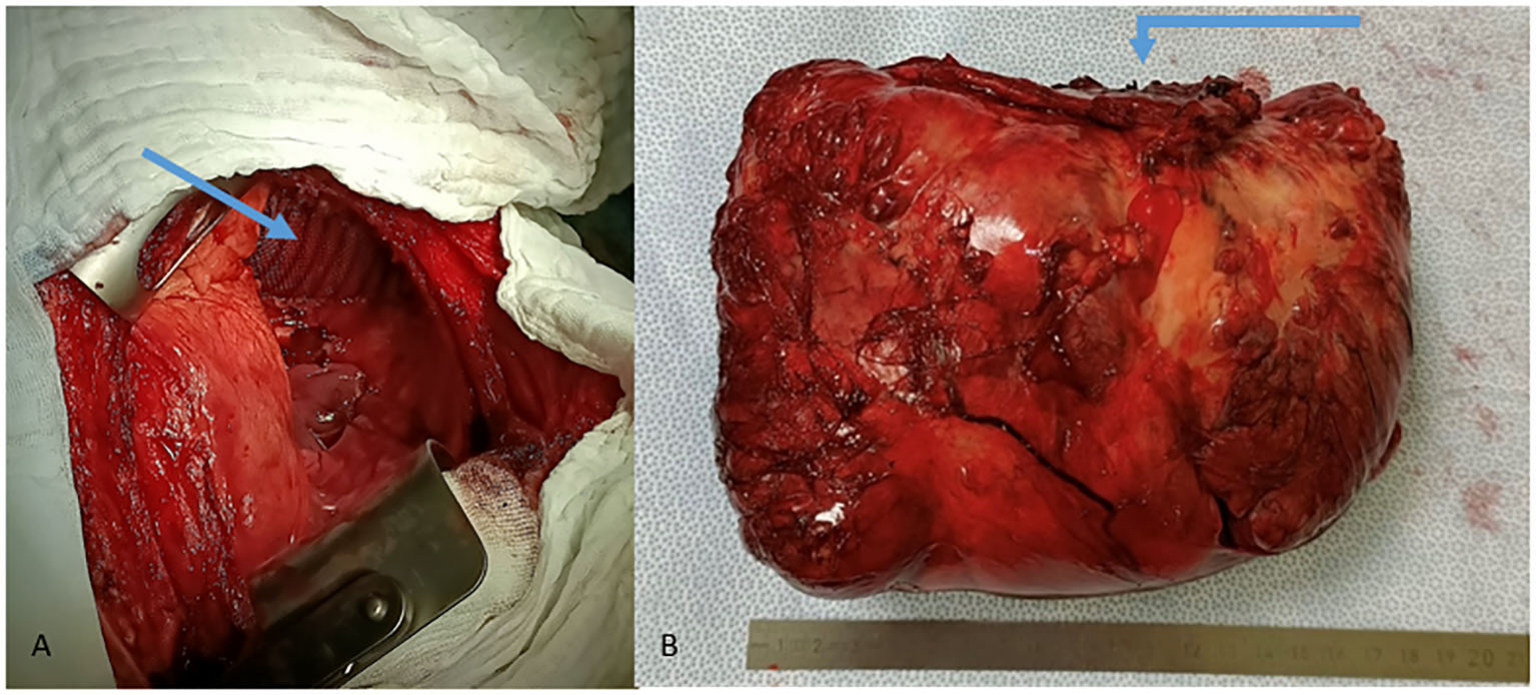
**(A)** Reconstruction of the diaphragm with Prolene mesh. **(B)** Partial excision of the diaphragm with surgical specimen.

The patient was admitted 2 days prior to surgery in order to control her blood glucose levels by administering intravenous glucose. During the perioperative preparation in a fasting state, she had no complications related to hypoglycemia. We first embolized the feeding artery arising directly from the aorta (**Figure 1**). We then proceeded to an en bloc resection with a diaphragmatic patch and extra-anatomic lung resection, followed by the reconstruction of the diaphragm with Prolene mesh through a left hemiclamshell incision (**Figure 2**).

## Results

The postoperative course was uneventful. The chest tube was removed on the second postoperative day, and the patient was discharged on the fourth postoperative day.

A partial remission of the hypoglycemia was witnessed after just the embolization. The complete resolution of the hypoglycemia was observed after the resection.

**Figure 3:**
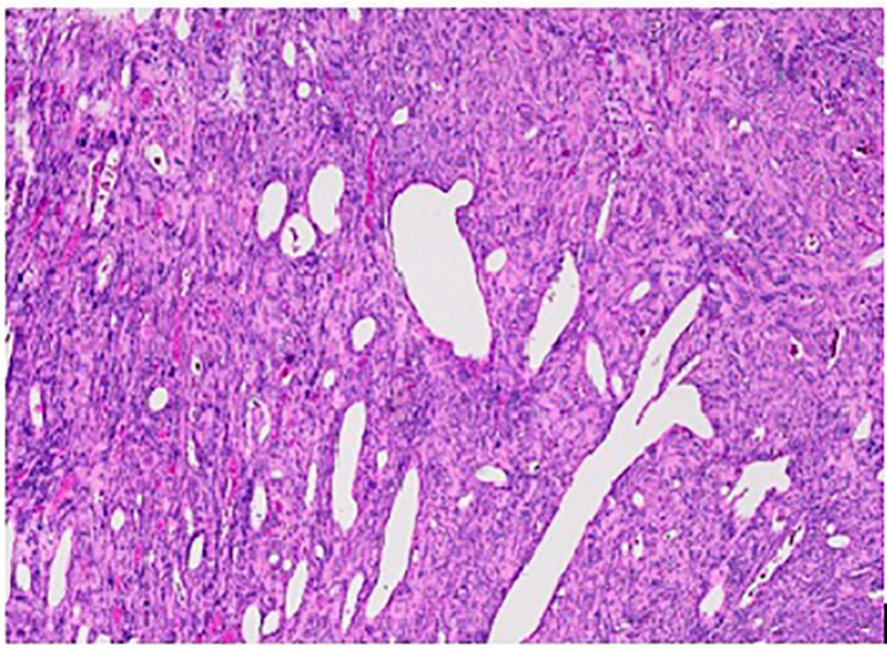
Microscopic view showing spindle cells (hematoxylin and eosin).

Macroscopic pathological examination showed a pSFT of 17.5 cm in maximal diameter and weight of 1,516 g (**Figure 2**), and microscopic view showed spindle cells (**Figure 3**) and clean margins. No features of malignancy in addition to the size and the associated NICTH were found: the immunohistochemical examination revealed low mitotic activity and Ki-67 positivity (<10%), no loss of CD34 activity, and no activity of S100 or CK116. Unfortunately, we were unable to find a laboratory team in Switzerland or elsewhere that could determine the plasma level of big insulin-like growth factor 2. At the outpatient follow-up 6 weeks after the operation, the patient reported no complications and a complete resolution of all her symptoms.

## Discussion

The patient is at the age with the peak frequency of discovery of NICTH. As often described in the literature, our patient experienced a delay in diagnosis of several weeks, necessitating two hospitalizations and finally an outpatient workup. Guiyan et al. reported a patient who presented with episodic confusion for 2 months before the diagnosis of NICTH (8). When faced with refractory hypoglycemia associated with low insulin and low C-peptide levels, it is important to look for other rare diagnoses and to involve an endocrinologist.

The contrast-enhanced CT scan revealed a blood-supplying artery coming directly from the aorta as the tumor’s sole pedicle (**Figure 1**). This allowed us to perform a preoperative embolization (**Figure 1**), which by itself ameliorated the blood glucose level and probably helped minimize perioperative blood loss. Yongsen et al. described a pSFT with a blood supply from an artery that also came directly from the aorta. This variant was identified in one case with the same anatomy and found in only two others in the literature (4). Kelin et al. described a case of giant pSFT and performed a preoperative embolization that permitted the safe removal of the tumor (1). This illustrates the importance of efficient planning of both embolization and the surgical approach.

Hospitalization during the fasting periods minimized the risk of complications due to severe hypoglycemia. In the postoperative course, the hypoglycemia was then quickly resolved, as in the case of Do Wan et al. (9), which reinforced our diagnosis of NICTH even before definitive histology was available. Their patient and the patient of Ahluwalia et al. (10) almost became comatose as a result of hypoglycemia due to pSFT (9). Surgery was the only solution. This shows how important it is to manage blood glucose properly.

Our patient’s tumor was benign. Its large size and the patient’s paraneoplastic symptoms were risk factors for malignant pSFT. However, whether the lesion was benign or malignant, its management would have been the same.

In accord with prior reports and facing a 15-cm tumor at the CT scan, we proceeded first to an embolization to increase the safety of our surgical management through a hemiclamshell incision. Histology confirmed the suspicion of pSFT with no malignant features upon immunohistochemical examination, and the return to normal glycemic levels is consistent with Doege–Potter syndrome. Post-operatively, she felt reassured once her blood sugar levels had stabilized, as she constantly had to eat small meals with the constant fear of hypoglycemia. We will conduct, as proposed in the literature and accepted by our multidisciplinary tumor board, a follow-up with a CT scan every 6 months for 2 years and then a CT scan every year to ensure that the tumor has not recurred. The first CT scans at 6 and 12 months showed no evidence of disease.

## Data Availability

The original contributions presented in the study are included in the article/Supplementary Material. Further inquiries can be directed to the corresponding author.
